# Ambient AI in primary care: an exploratory mixed methods survey of UK general practitioners

**DOI:** 10.1136/bmjhci-2025-101847

**Published:** 2026-07-01

**Authors:** Charlotte Blease, Anna Kharko, Carolina Garcia Sanchez, David Navarro, Brian McMillan, Jens Gaab, Cosima Locher, Enrico Coiera

**Affiliations:** 1 Department of Women's and Children’s Health, Uppsala University, Uppsala, Sweden; 2 Centre for Primary Care and Health Services Research, The University of Manchester, Manchester, UK; 3 Centre for Health Informatics, Australian Institute for Health Innovation, Macquarie University, Sydney, New South Wales, Australia; 4 The University of Manchester, Manchester, UK; 5 Department of Clinical Psychology and Psychotherapy, Faculty of Psychology, University of Basel, Basel, Switzerland; 6 Clinical Psychology and Psychosomatics, Faculty of Psychology, University of Basel, Basel, Switzerland; 7 Centre for Health Informatics, Australian Institute of Health Innovation, Macquarie University, Sydney, New South Wales, Australia

**Keywords:** Artificial intelligence, Primary Health Care, Informatics, Documentation, Electronic Health Records

## Abstract

**Objective:**

To examine UK general practitioners’ (GPs) adoption of ambient artificial intelligence (AI) scribes and to assess user-reported error rates, workflow impact and consent practices in primary care.

**Methods:**

We conducted a nationwide online mixed-methods survey of GPs recruited via Doctors.net.uk. Detailed analyses of use, errors and workflow impact focused on current users of ambient AI scribes.

**Results:**

In August 2025, of 1003 respondents, 14% (n=141) reported current use of ambient AI scribes, 39% (n=396) intended to adopt them soon and 46% (n=466) had no plans to use them. Among users (n=141), Heidi Health predominated (86%). Most reported efficiency gains: 80% (n=112) reported reduced time spent on documentation and 70% (n=99) reduced cognitive load. Documentation quality was judged positively, with 55% (n=78) rating outputs as better than standard notes. Errors were common but usually minor: 32% (n=45) reported errors often/always, including 14% (n=20) with significant-to-critical implications. Errors were most frequent in multiparty consultations (38%), complex histories (35%) and non-English encounters (31%). Consent practices varied: 63% (n=89) routinely sought consent, with ≤10% of patients declining. Free-text responses (21% of users) highlighted benefits for workflow, alongside concerns about accuracy, ethics and system integration.

**Discussion:**

Findings suggest that ambient AI scribes deliver meaningful efficiency gains and improved perceived documentation quality, but introduce non-trivial risks related to accuracy, equity and medicolegal accountability. The uneven performance in complex and multilingual consultations raises particular concerns about potential exacerbation of healthcare disparities.

**Conclusion:**

Ambient AI scribes are already in use across UK primary care. Proactive regulation, consistent consent practices and independent evaluation, including patient perspectives, are urgently needed to ensure safe, equitable and sustainable implementation.

**WHAT IS ALREADY KNOWN ON THIS TOPIC**

- Ambient artificial intelligence scribes are marketed as tools to ease documentation and reduce burnout, but evidence on real-world uptake and general practitioner (GP) perspectives in the UK is sparse.

**WHAT THIS STUDY ADDS**

- This survey shows early but growing adoption, with GPs reporting clear efficiency gains but also accuracy concerns, especially in complex and multilingual consultations.

**HOW THIS STUDY MIGHT AFFECT RESEARCH, PRACTICE OR POLICY**

- Findings highlight the need for regulation, consistent consent practices and prospective studies, including patient perspectives, to guide safe and equitable implementation.

## Introduction

 Ambient artificial intelligence (AI) scribes, also referred to as ‘digital scribes’^1^ or ambient voice technology (AVT), are emerging tools designed to passively capture clinician–patient conversations and automatically generate documentation within electronic health records.^2^ This is a rapidly expanding market which may reduce the documentation burden on clinicians, thereby addressing one of the major drivers of burnout in primary care.

Despite this promise, their use raises important challenges.^3 4^ Concerns include accuracy and reliability of transcribed notes, risks of errors or omissions in documentation, medicolegal liability, patient consent and the potential loss of nuance in clinical communication.^5^ Furthermore, biases in speech recognition and summarisation may result in systematic disadvantages for certain groups of patients, such as those with strong regional or foreign accents or individuals with speech disorders. The rapid pace of product development may also give rise to uncertainty about consistency, integration into existing record systems and implications for professional practice and regulation, particularly as governance frameworks continue to evolve.

Although interest in ambient AI scribes is growing, there is little empirical evidence on how widely they are being adopted in frontline general practice and how they are perceived by clinicians who use them.^6^ Few studies have directly explored the lived experiences, uptake and opinions of doctors regarding these tools. Given the apparent interest and rapid adoption in primary care settings, this study seeks to fill that gap by examining general practitioners’ (GPs) use of and attitudes towards ambient AI scribes in the UK.

## Methods

### Survey design and recruitment

We conducted a nationwide, anonymous, web-based mixed-methods survey of UK GPs via Doctors.net.uk, the largest online professional network for UK doctors. Membership is free but requires General Medical Council (GMC) verification; participation was restricted to verified GPs. Platform authentication confirmed professional status and prevented duplicate submissions. At the time of the study, Doctors.net.uk had 254 741 members, representing approximately two-thirds of the UK’s 379 208 registered physicians. The survey was incorporated into Doctors.net.uk’s regular ‘Omnibus’ series, which explores current medical topics and samples 1000 clinicians each month, a size comparable to other healthcare workforce surveys.^7^ The research team previously collaborated with the platform on similar GP surveys.^8 9^

Between 7 and 27 August 2025, invitations were distributed via homepage dashboard adverts and targeted emails to GPs who had opted into research communications. During this period, 9024 GPs logged into Doctors.net.uk while the survey was live. Stratified random sampling was used to ensure national coverage, with regional strata informed by GMC Data Explorer demographics. Because participation depended on platform engagement and voluntary response, the sample should be considered a convenience sample and may over-represent digitally engaged GPs; findings therefore describe adoption patterns rather than population prevalence.

### Survey instrument and administration

This survey follows the format of previous studies we have conducted into UK GPs’ experiences and opinions with AI tools.^8 9^ We developed an anonymous, ~5 min questionnaire defining ambient AI scribes as background systems that capture consultation dialogue and automatically generate clinical documentation (online supplemental appendix 1). A screening item restricted the main survey to current users, while non-users were asked about intended near-term adoption.

The survey included eight core closed-ended items (with conditional follow-ups), seven demographic questions and one optional free-text prompt. Items covered products used, transcription errors and corrections, subgroup variation, medicolegal concerns, workload, burnout, documentation time, note quality and consent practices. All closed-ended questions were mandatory; the free-text item was optional. All measures were self-reported and not independently verified. The optional free-text item was intended to provide brief contextual comments rather than in-depth qualitative accounts.

The instrument was piloted with five UK-based GPs recruited via professional contacts, none of whom participated in the main survey. Feedback addressed minor wording ambiguities, clarification of the definition of ambient AI scribes and survey length. Items and response options were refined accordingly and the survey was streamlined to enable completion within 3–5 min. Design and reporting followed the Checklist for Reporting Of Survey Studies guidelines (online supplemental appendix 2).

### Quantitative component

Participant characteristics and responses to survey opinion items were summarised through descriptive statistics. To analyse whether users and non-users of ambient AI scribes differed in gender, age, role or practice size, a χ^2^ of independence test was carried out for each variable. If significant, it was followed by a post hoc two-proportion z-test, where one variable level was tested against all others. Bonferroni-adjusted p value was used. Given our non-random sample, to analyse whether differences with GPs in the GMC Registry, a χ^2^ goodness-of-fit test was used. For key proportions, CI 95% were calculated using the Wilson score method for binomial proportions. Given the non-probability sampling, CIs describe precision around sample estimates rather than population prevalence. Significance levels were pre-set to p<0.05 for all tests. Analyses were carried out by AK in JASP (V.0.95.1.0) and jamovi (V.2.6.2). Tables and figures were created by AK.

### Qualitative component

The survey included a single optional free-text item (‘Please add any comments about the topic or the survey (1–2 brief comments)’). Trivial responses (eg, ‘none’, ‘N/A’) were removed. Given the limited volume and brevity of responses—typically short phrases or one to two sentences—full inductive thematic analysis was not appropriate. Instead, a descriptive content-focused approach was used to identify and summarise recurrent issues raised by respondents. This approach is commonly used for optional free-text survey data and allows qualitative insights to complement and contextualise quantitative findings.^10^ Accordingly, qualitative findings should be interpreted as illustrative rather than exhaustive.

CB reviewed the data, developed descriptive codes and iteratively refined them; responses containing multiple ideas received multiple codes. First-order codes were grouped into higher-order categories to summarise recurrent patterns. CGS independently repeated this process, and minor discrepancies were resolved through discussion until consensus was reached, with no formal arbitration required given the rudimentary, descriptive rather than interpretive, nature of the coding through discussion. We emphasise that dual coding was used to support interpretive consistency, not to establish statistical reliability or thematic representativeness.

## Results

### Quantitative analysis

#### Sample characteristics and adoption

Of the 2855 who received an email invitation, 62% (n=1776) opened it, 38% (n=676) clicked on the survey link and 21% (n=601) completed the survey. This resulted in a response rate of 21% among the invited sample (calculated as 601 ÷ 2855 × 100). In addition, 402 respondents completed the survey via an embedded homepage dashboard advertisement on Doctors.net.uk; a denominator for homepage impressions or click-throughs was not available, so a response rate for this recruitment route could not be calculated. Together, these sources yielded a total sample of 1003 respondents. Of them, 46% (n=466; 95% CI 43.4 to 49.6) reported that they neither used ambient AI scribes nor intended to in the near future, 39% (n=396; 95% CI 36.5 to 42.6) reported that they were not currently using them but intended to do so and 14% (n=141; 95% CI 12 to 16.3) reported they used these tools. In the whole sample, half were women (n=503) and nearly half were aged 35–45 years old (45%, n=452), see table 1.

**Table 1:** Respondent characteristics of GPs who were users and non-users of ambient AI

	Users (n=141)	Non-users (n=862)	Whole sample (n=1003)
Gender			
Woman	64 (45.4%)	439 (51%)	503 (50.1%)
Man	74 (52.5%)	407 (47.2%)	481 (48%)
Other	–	1 (0.1%)	1 (0.1%)
Preferred not to say	3 (2.1%)	15 (1.7%)	18 (1.8%)
Age			
35 years or younger	15 (10.6%)	88 (10.2%)	103 (10.3%)
36–45 years	62 (44%)	287 (33.3%)	349 (34.8%)
46–55 years	50 (35.5%)	321 (37.2%)	371 (37%)
56 years or older	14 (9.9%)	166 (19.3%)	180 (17.9%)
Role			
GP partner or principal	76 (53.9%)	350 (40.6%)	426 (42.5%)
Salaried GPs	52 (36.9%)	332 (38.5%)	384 (38.3%)
Locum GPs	7 (5%)	146 (17%)	153 (15.3%)
GP Registrar	6 (4.3%)	34 (3.9%)	40 (4%)
GP practice size			
Up to 5000 patients	6 (4.3%)	105 (12.2%)	111 (11.1%)
5001–7500 patients	19 (13.4%)	131 (15.2%)	150 (15%)
7501–10 000 patients	28 (19.9%)	175 (20.3%)	203 (20.2%)
10 001–12 500 patients	24 (17%)	148 (17.2%)	172 (17.1%)
12 501 patients or more	64 (45.4%)	303 (35.1%)	367 (36.6%)

Percentages are calculated based on column total.

AI, artificial intelligence; GPs, general practitioners.

The sample differed from the GMC Registry on gender: there were significantly fewer women in our sample, χ^2^(1,984) = 19.38, p<0.001, Cramer’s V=0.14, but did not differ on regional distribution, see online supplemental appendix 3.

Among current users of ambient AI scribes (n=141), fewer GPs aged≥56 reported using ambient AI (−9%, adjusted p=0.03) see online supplemental appendix 3. Use did not differ by gender (χ²(1,984)=1.48, p=0.224), but significant differences were observed for age (χ²(3,1003)=9.97, p=0.019, Cramer’s V=0.10), role (χ²(3,1003)=16.55, p<0.001, Cramer’s V=0.13) and practice size (χ²(4,1003)=10.6, p=0.031, Cramer’s V=0.10) (see online supplemental appendix 3). Users were more likely to be GP partners or principals (+13%, adjusted p=0.012) and less likely to be locum GPs (−12%, adjusted p=0.001). Users were also less likely to report working in smaller practices with up to 5000 patients (−8%, adjusted p=0.027).

Among current users of ambient AI scribes (n=141), *Heidi Health* was the most popular AI scribe used by 86% of GPs, see table 2.

**Table 2:** Ambient AI scribes used by GPs (n=141)

	Users (n=141)
Heidi Health	122 (86.5%)
Accurx Scribe	18 (12.8%)
i-Scribe	2 (1.4%)
Suki AI	1 (0.7%)
DeepScribe	1 (0.7%)
Anima Scribe	3 (2.1%)
Lexacom Echo	2 (1.4%)
Meta AI	1 (0.7%)
RingCentral AI Transcription	1 (0.7%)
Tandem AI	1 (0.7%)
Tortus	1 (0.7%)
Unspecified	1 (0.7%)

Scribes that were listed but received no votes: Augnito Spectra, Dragon Ambient eXperience, Heparin Write, HepianScribe, Lyrebird Health, Tali.AI. The survey item was multiple choice; total count exceeds user count. Also, respondents could select more than one ambient AI scribe; percentages therefore exceed 100%.

AI, artificial intelligence; GPs, general practitioners.

Two-thirds of GPs (63%, n=89; 95% CI 54.9 to 70.6) reported that they routinely obtained consent from patients before using AI during the consultation. Among them (n=89), most GPs reported that 10% of all patients or fewer declined consent (89%, n=79; 95% CI 80.5 to 93.8), see online supplemental appendix 3.

#### Workflow impact

As seen in table 3, among ambient AI users (n=141), a third of respondents reported working 30–40 hours per week and most (85%, n=122) reported seeing at least 26 patients daily. 27% (n=38) reported experiencing varying degrees of burnout. Among respondents, most reported that ambient AI decreased cognitive workload during consultation (70%, n=99) and decreased time spent creating documentation (80%, n=112).

**Table 3:** Workload and impact of ambient AI scribes on it among current users (n=141)

	Users (n=141)
Hours worked per week	
Fewer than 10 hours	2 (1.4%)
10–20 hours	16 (11.3%)
20–30 hours	28 (19.9%)
30–40 hours	45 (31.9%)
40–50 hours	35 (24.8%)
More than 50 hours	15 (10.6%)
Patients seen per day	
0	–
1–5	1 (0.7%)
6–10	4 (2.8%)
11–15	5 (3.5%)
16–20	10 (7.1%)
21–25	29 (20.6%)
26–30	63 (44.7%)
31 or more	29 (20.6%)
Self-rated level of burnout	
I enjoy my work. I have no symptoms of burnout.	21 (14.9%)
Occasionally I am under stress, but I don’t feel burned out.	82 (58.2%)
I am definitely burning out and have one or more symptoms of burnout.	24 (17%)
The symptoms of burnout that I’m experiencing won’t go away.	9 (6.4%)
I feel completely burned out and often wonder if I can go on.	5 (3.5%)
Impact of ambient AI on cognitive workload	
Significantly decreased	28 (19.9%)
Somewhat decreased	71 (50.4%)
No change	39 (27.7%)
Somewhat increased	3 (2.1%)
Significantly increased	–
Impact of ambient AI on time spent on documentation	
Greatly decreased	42 (29.8%
Slightly decreased	70 (49.6%)
No change	18 (12.8%)
Slightly increased	10 (7.1%)
Greatly increased	1 (0.7%)

Refer to online supplemental appendix 1 for the exact wording of the survey items.

AI, artificial intelligence.

Among current users of ambient AI scribes (n=141), over half (55%, n=78; 95% CI 47 to 63.3) rated AI-generated documentation as *‘Good (better than standard medical documentation, requiring minimal revision)’*, see online supplemental appendix 3.

#### Errors and verification practices

**Figure 1:**
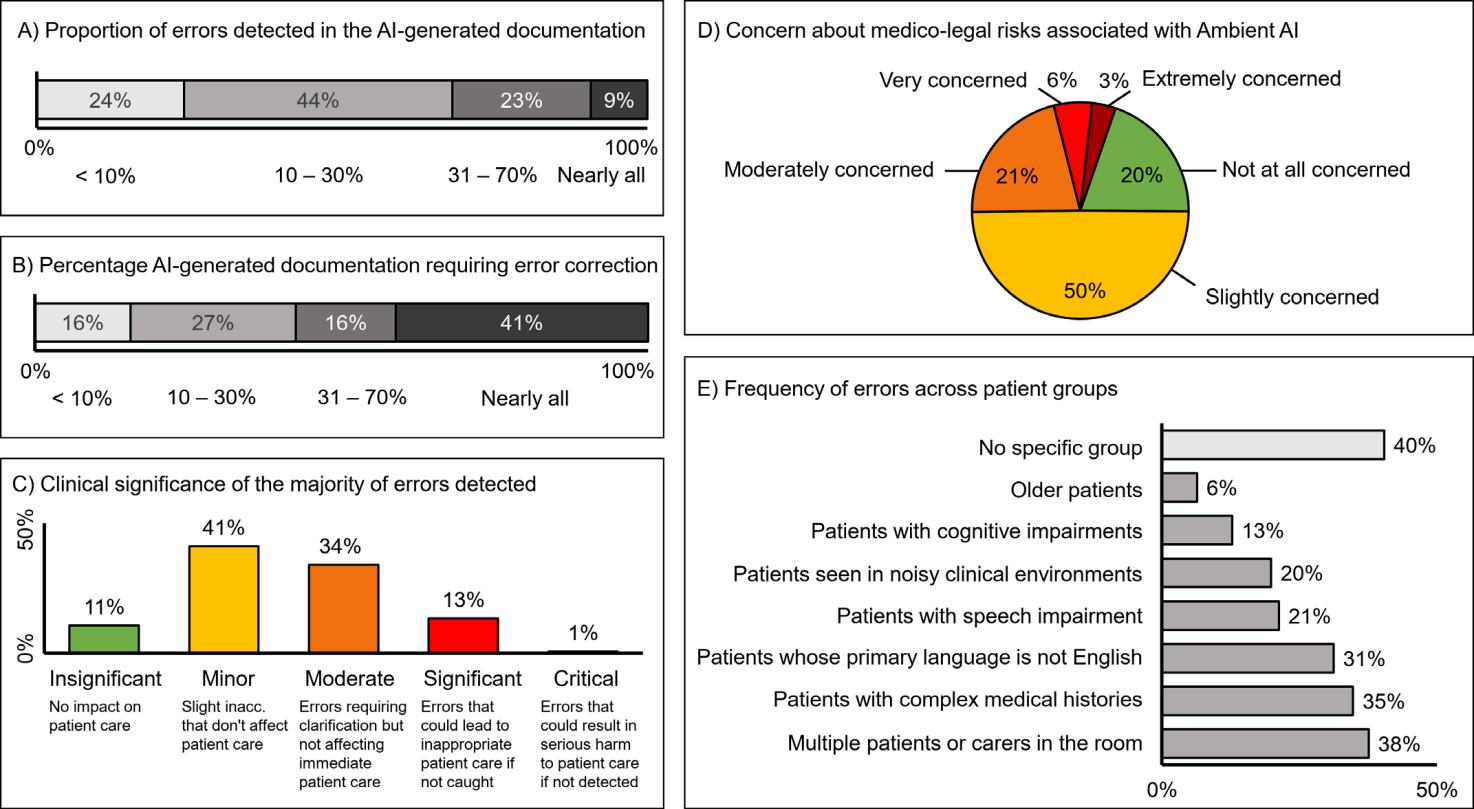
(A–E) Errors and medicolegal risks associated with ambient AI documentation among current users (n=141). AI, artificial intelligence.

At the same time, almost half of GPs (44%) reported that they find errors in 10–30% of the documentation and a similar proportion (41%) correct nearly all generated documentation with 16% reporting correcting 10% or less, see figure 1A–E.

Further evaluating the errors, half of GPs who used these tools (52%, n=73) considered them *‘insignificant’* or *‘minor’* and not affecting patient care.

#### Consent and medicolegal perceptions

In our sample, half of GP adopters of ambient AI (50%, n=70) were *‘slightly concerned’* about the medicolegal risks associated with using AI scribes. Consultations with specific patient groups generated more ambient AI errors, for example, when there are multiple patients or carers present in the room (38%, n=53) and patients with complex medical histories (35%, n=49).

### Qualitative analysis

Of the 141 respondents, 29 (21%) provided free-text comments, generating a total of 689 words after data cleaning (see Raw data, online supplemental appendix 4). Most entries were concise, often comprising short phrases or 1–3 sentences. Through inductive coding and iterative analysis, four overarching categories of views on AI scribe use in general practice were identified: (1) perceived benefits in workflow and patient care; (2) accuracy and reliability concerns; (3) ethical and professional considerations; and (4) future expectations and system-level challenges. Quotes are presented verbatim and anonymised (participant number, gender, age in parentheses). Responses reflected cautious optimism: while AI scribes were valued for reducing workload and improving patient focus, concerns about accuracy, professional identity and system integration highlighted the need for continued refinement and oversight. See table 4.

**Table 4:** Example quotes for emerging categories

Category	Description	Example quotes
#1	Perceived benefits in workflow and patient care	“helpful and time saving*”* [#131, F, 36–45] “very useful” [#54, M, 46–55] “concentrate on the patient rather than the keyboard” [#133, M, 56 or over]
#2	Accuracy and reliability concerns	“miss important bits” [#67, M, 56 or over] “It makes every consult look the same which makes the review of notes less meaningful” [#49, F, 56 or over] “The notes don’t capture the uncertainty and art of general practice” [#10, F, 36–45]
#3	Ethical and professional considerations	“I don't ask consent because it is just a scribing tool. I have never historically asked consent to use a Dictaphone to dictate patient referral letters either - there is no difference” [#73, F, 45–55] “there are notices throughout the practice, and on consulting room doors and consulting room desks indicating that AI scribes are being used” [#91, M, 56 or over] “…directly as a result of this being pulled as a tool to support GPs I am likely to have to quit working as a GP in the NHS” [#15, M, 46–55]
#4	Future expectations and system-level challenges	“a step in the right direction” [#31, M, 36–45] “It is great but needs vigilant monitoring to correct errors” [#15, M, 46–55] “I expect it will improve further and we should embrace it” [#66, F, 35–45]

Example quotes chosen.

AI, artificial intelligence; F, female; GP, general practitioner; M, male; NHS, National Health Service.

## Discussion

### Summary of main findings

Administered in August 2025, this exploratory online survey (n=1003) is, to our knowledge, the largest UK study of GPs to assess current use and intended adoption of ambient AI scribes. Almost one in seven had used these tools (141/1003; 14%), with a further 39% intending to adopt them soon (396/1003); among users, Heidi Health predominated (121/141; 86%). Exploratory subgroup analyses among users (n=141) found no gender differences, but uptake varied across workforce segments. Adoption was lower among older GPs and locums and higher among GP partners/principals and those in larger practices. These patterns may reflect differences in autonomy, resources and incentives, but should be interpreted cautiously given the modest sample size.

These characteristics also affect interpretation of reported benefits. Early adopters may have greater administrative support, digital infrastructure, or flexibility in workflow redesign than smaller or locum-led practices. They may also be more positively biased towards technology resulting in a halo effect when evaluating technology performance. Reported efficiency gains and manageable error rates may therefore reflect favourable implementation contexts rather than broader conditions across general practice. As such, our results should be interpreted as an early-adoption snapshot rather than expected performance across the wider workforce.

Among users (n=141), error detection was common but usually not severe: 32% (45/141) reported errors often or always, yet 86% (121/141) judged them insignificant-to-moderate and 14% (20/141) significant-to-critical; 57% (81/141) corrected errors often or almost always.

Also, among users (n=141), perceived error risk was highest in multiparty consultations (38%), complex histories (35%), non-English primary language (31%), speech impairment (21%), noisy environments (20%) and cognitive impairment (13%)—patterns that may entrench the inverse care law, with those most in need again underserved. Several mechanisms may underlie these disparities. Speech recognition and natural language processing systems perform less reliably with accented, non-native, code-switched or multispeaker dialogue. Complex or lengthy histories may also strain current summarisation models, increasing risks of omission, misattribution or loss of clinical nuance. If unaddressed, these limitations could disproportionately affect patients already facing communication and documentation barriers, thereby exacerbating existing health inequities.

These patterns also affect how the technology should be used in practice. Ambient AI performance that varies by consultation type requires greater awareness among clinicians. For example, in consultations involving interpreters, several participants or complex clinical narratives, additional checking—and sometimes avoidance of the tool—may be appropriate. Services adopting ambient AI should therefore examine accuracy across patient groups rather than relying on overall aggregate performance and patients should be informed and able to opt out without disadvantage. Otherwise, documentation efficiencies for clinicians may come at the cost of reduced reliability for those who already face communication barriers.

Workload signals were favourable among those who adopted these tools: 80% (112/141) reported reduced time spent on documentation (30% greatly; 50% slightly) and 70% (99/141) reported decreased cognitive load (20% significant; 50% somewhat); 28% noted no change and only 2% an increase. Medicolegal concerns were limited—9% (13/141) were very/extremely concerned. Consent practices varied: 63% (89/141) reported routinely seeking patient consent; among these, 89% (79/89) said ≤10% of patients declined. Overall, as supported by the free text comments, GPs reported meaningful efficiency gains alongside manageable, though non-trivial, error, equity and governance concerns.

At first sight these findings appear inconsistent: errors were commonly detected and frequently corrected, yet concerns about medicolegal risk were limited and most clinicians still reported time savings. One explanation is that clinicians are not treating the output as autonomous documentation but as a draft requiring verification. In this workflow, the efficiency gain may arise from editing a structured summary rather than composing notes from scratch. Time savings may therefore occur alongside ongoing correction, with GPs reviewing and approving the final record. These findings are thereby more consistent with assisted documentation than with replacement of clinical documentation tasks.

### Comparison with prior work

Our findings align with a qualitative study of 22 pilot physicians showing reduced documentation burden and cognitive load, but recurring barriers in non-English encounters.^11^ They also accord with a US pre–post study in ambulatory care reporting meaningful reductions in clinician burnout and signs of improved professional fulfilment with ambient AI.^12^ Together, the evidence indicates efficiency and well-being gains alongside typically low-severity errors, with weaker performance in multilingual and complex consultations. Clinicians in our sample judged ambient AI scribes to improve documentation quality, even while acknowledging detectable errors. These assessments were subjective and occurred alongside frequent correction, so they should not be interpreted as objective quality improvement. Notably, most errors were rated insignificant-to-moderate, with only a minority judged significant-to-critical. This sits alongside prior UK GP survey evidence in which roughly 60% anticipated that patients would find significant errors in their records^13^ and US patient data showing that one in five note-readers reported a mistake and 40% of those considered it serious.^14^ Against that backdrop, our pattern of responses suggests that, while errors persist, the error burden with ambient AI may be lower than with prevailing documentation approaches—though direct evaluations are needed to confirm this.

Most users in this sample reported using a single platform (Heidi Health), limiting generalisability. Error rates, workflow effects and user experiences may differ across systems with varying models, training data, customisation and EHR integration. Head-to-head comparative evaluations are needed to determine whether these findings extend across platforms. Beyond this, our own recent studies have documented an uptick in the availability and use of generative AI tools in clinical settings in the UK^8 9^; ambient AI scribes appear to be part of this wider diffusion trend.

In April this year, National Health Service England (NHSE) issued guidance on the use of digital scribes in clinical settings.^15^ This guidance places key responsibilities on users of this technology, namely that they need to: (1) assign a clinical safety officer to identify potential risks, (2) conduct a Data Protection Impact Assessment, (3) ensure the tool is appropriately integrated into existing systems, (4) ensure the tool meets existing data security and medical device regulations and that staff are appropriately trained and (5) implement ongoing monitoring of the tool’s performance.^15^ Due to concerns about patient safety, NHSE’s chief clinical information officer wrote to NHS organisations in June this year to warn that staff must immediately stop using tools that had not been registered as a class I medical device with the UK’s Medicines and Healthcare Products Regulation Agency.^16^ This letter stated that a national plan to standardise AVT deployment in England was being developed and that further guidance would be published to ensure automatic deletion of patient data acquired by AVT systems.^16^ These integration and governance challenges represent forward-looking considerations informed by the evolving regulatory landscape, rather than empirical findings of this study. We emphasise that this study did not assess regulatory status of individual products and cannot determine whether tools reported by respondents met these requirements.

Similar warnings about avoidance of use of unregulated generative AI have been issued by health departments in Australia.^17^ The Australian professional regulator Ahpra also explicitly embeds seeking informed patient consent prior to using a scribe in its core professional code of conduct for clinicians.^18^ However, it is still unclear whether clinicians in the UK are obligated to seek patient consent for the use of ambient AI scribes.^15^ Beyond consent requirements, current regulation in both the UK and Australia places responsibility for the accuracy of the clinical record on the clinician, not the technology. Use of AI scribes does not alter this legal accountability; clinicians remain liable for the content of the record.

Regulatory uncertainty does not remove the ethical relevance of informing patients about the use of ambient AI tools. Given that most patients were reported to consent when asked, routine notification and the opportunity to decline appear feasible in practice. We caution that failure to secure consent risks undermining transparency and trust.

### Strengths and limitations

This exploratory study delivers a timely UK snapshot of GP experience with ambient AI scribes. To our knowledge, it is the largest survey to date (n=1003; August 2025), capturing both current users and near-term adopters. However, the study has several limitations. The convenience, non-random sample and self, online administration limited generalisability and may have been compromised by self-selection, recall bias and non-response bias; all measures are self-report rather than linked to objective metrics. Recruitment via a professional platform may also have over-represented digitally engaged GPs and therefore should be interpreted as an early-adopter snapshot rather than population-representative estimates.

Recruitment occurred via email invitation and homepage advertisement; response rates were calculable only for the email sample. The absence of a denominator for homepage exposure precludes estimation of response rate for that route, and homepage respondents may differ systematically (eg, greater engagement or topic interest), introducing potential selection bias. The modest sample of current users limited power to examine determinants of uptake and precluded robust subgroup or tool-specific analyses of error patterns. Outcomes were also self-reported; we lacked objective or independent measures of error rates and workflow impact. Finally, the qualitative component was confined to a single optional free-text response, which restricted depth and may have failed to capture divergent views.

Given rapid product evolution, these findings may quickly date. Ongoing, stratified UK sampling is needed to monitor adoption and experience. Future research should be prospective and multisite, incorporating objective workflow and safety metrics. Patient perspectives—including on consent, trust and acceptability—must also be systematically examined to ensure a balanced ethical and clinical assessment of ambient AI scribes.

## Conclusion

This convenience survey of UK GPs provides the largest snapshot to date of ambient AI scribe use in frontline practice. Although current uptake is modest (14%), anticipated near-term adoption (39%) signals rapid diffusion. Reported benefits include reduced documentation time and cognitive load, but clinically significant errors and concerns in complex or multilingual consultations persist. Ambient AI scribes may ease workload, yet risk widening inequities and introducing medicolegal harms without robust governance. While we acknowledge that this is a convenience sample, and should be interpreted accordingly, we recommend that proactive regulation, consistent consent practices and systematic safety monitoring are needed. Notably, 37% of current users in this sample did not routinely seek patient consent—a finding with immediate clinical and regulatory implications. Prospective, multisite studies incorporating objective workflow, safety and patient-reported outcomes will be essential to define their long-term role in primary care.

## Supplementary material

10.1136/bmjhci-2025-101847 online supplemental appendix 1

10.1136/bmjhci-2025-101847 online supplemental appendix 2

10.1136/bmjhci-2025-101847 online supplemental appendix 3

10.1136/bmjhci-2025-101847 online supplemental appendix 4

## Data Availability

All data relevant to the study are included in the article or uploaded as an online supplemental information.

## References

[1] Coiera E, Kocaballi B, Halamka J (2018). The digital scribe. NPJ Digit Med.

[2] Haberle T, Cleveland C, Snow GL (2024). The impact of nuance DAX ambient listening AI documentation: a cohort study. J Am Med Inform Assoc.

[3] Blease C, Torous J (2023). ChatGPT and mental healthcare: balancing benefits with risks of harms. *BMJ Ment Health*.

[4] Blease C (2024). Open AI meets open notes: surveillance capitalism, patient privacy and online record access. *J Med Ethics*.

[5] Ha E, Choon-Kon-Yune I, Murray L (2025). Evaluating the usability, technical performance, and accuracy of artificial intelligence scribes for primary care: competitive analysis. JMIR Hum Factors.

[6] Ma SP, Liang AS, Shah SJ (2025). Ambient artificial intelligence scribes: utilization and impact on documentation time. J Am Med Inform Assoc.

[7] Thornton N, Binesmael A, Horton T (2024). AI in health care: what do the public and NHS staff think?. https://www.health.org.uk/publications/long-reads/ai-in-health-care-what-do-the-public-and-nhs-staff-think.

[8] Blease C, Garcia Sanchez C, Locher C (2025). Generative artificial intelligence in primary care: qualitative study of UK general practitioners’ views. J Med Internet Res.

[9] Blease CR, Locher C, Gaab J (2024). Generative artificial intelligence in primary care: an online survey of UK general practitioners. BMJ Health Care Inform.

[10] Joffe H, Yardley L, Marks DF, Yardley L (2004). Research methods for clinical and health psychology.

[11] Shah SJ, Crowell T, Jeong Y (2025). Physician perspectives on ambient AI scribes. JAMA Netw Open.

[12] Misurac J, Knake LA, Blum JM (2025). The effect of ambient artificial intelligence notes on provider burnout. Appl Clin Inform.

[13] Blease CR, Kharko A, Dong Z (2024). Experiences and opinions of general practitioners with patient online record access: an online survey in England. *BMJ Open*.

[14] Bell SK, Delbanco T, Elmore JG (2020). Frequency and types of patient-reported errors in electronic health record ambulatory care notes. JAMA Netw Open.

[15] NHS England (2025). Guidance on the use of AI-enabled ambient scribing products in health and care settings. https://www.england.nhs.uk/long-read/guidance-on-the-use-of-ai-enabled-ambient-scribing-products-in-health-and-care-settings/.

[16] Moberly T (2025). Doctors must stop using unregistered AI scribe tools, says NHS England. *BMJ*.

[17] Safer Care Advisory (2023). Health service use of unregulated artificial intelligence (AI). Melbourne, Victoria, Australia. https://www.safercare.vic.gov.au/sites/default/files/2023-07/Advisory%20-%20ChatGPT%20and%20Generative%20AI%20July%202023%20FINAL.pdf.

[18] Australian Health Practitioner Regulation Agency Meeting your professional obligations when using artificial intelligence in healthcare. https://www.ahpra.gov.au/Resources/Artificial-Intelligence-in-healthcare.aspx.

