# Supplementary material for: Ambient AI in primary care: an exploratory mixed methods survey of UK general practitioners
Source: BMJ Health Care Inform. 2026 Jul 1;33(1):e101847. doi: 10.1136/bmjhci-2025-101847 (PMC13331185; doi:10.1136/bmjhci-2025-101847)
Supplement: online supplemental appendix 1 [file bmjhci-33-1-s001.docx]

# Appendix 1: Survey

## Survey on the Use of Ambient (AI) Scribes in Primary Care

#### **Introduction**

The purpose of this study is to explore general practitioners' experiences and perceptions of using **ambient AI scribes** for clinical documentation in electronic health records (EHRs). Ambient AI scribes are advanced AI tools that operate in the background during clinical encounters, passively capturing conversations and generating documentation such as progress notes, referral letters, and other clinical records without requiring direct input or activation.

We are interested in your experiences and opinions about these tools. Your responses will help us assess their safety, efficiency, and quality.This survey is anonymous and will take approximately 5 minutes to complete.

### **Screening**

**Scr 1 Do you currently use ambient AI scribes to assist with visit documentation in the EHR?**

- ☐ Yes
- ☐ No but I intend to use ambient AI scribes in the near future (If no, please exit the survey. Thank you!)
- ☐ No, and I do not intend to use ambient AI scribes in the near future (If no, please exit the survey. Thank you!)

### **Demographic Questions**

**Current Employment**

The questions in this section are about your current employment.

**Dem1 - How many hours do you work per week (including time spent seeing patients, home visits, administrative tasks, etc.)?**

- ☐ Fewer than 10 hours
- ☐ 10-20 hours
- ☐ 20-30 hours
- ☐ 30-40 hours
- ☐ 40-50 hours
- ☐ More than 50 hours

**Dem2 - On average, how many patients do you see per day?**

- ☐ 0
- ☐ 1 - 5
- ☐ 6 - 10
- ☐ 11 - 15
- ☐ 16 - 20
- ☐ 21 - 25
- ☐ 26 - 30
- ☐ 31+

**S1 - Which of the following best describes your role?**

- ☐ GP Partner / Principal
- ☐ Salaried GP
- ☐ Locum GP
- ☐ GP Registrar

**S2 - Where are you currently practicing?**

- ☐ London
- ☐ South West
- ☐ South East
- ☐ West Midlands
- ☐ East Midlands
- ☐ East of England
- ☐ Yorkshire and Humber
- ☐ North East
- ☐ North West
- ☐ Scotland
- ☐ Wales
- ☐ Northern Ireland
- D1 - Are you...
- ☐ Male
- ☐ Female
- ☐ Other
- ☐ Prefer not to say

**D2 - Are you...**

- ☐ 35 or under
- ☐ 36 to 45
- ☐ 46 to 55
- ☐ 56 or over

**D3 - Which of the following best describes the area where your practice is based?**

- ☐ NHS North Central London ICB
- ☐ NHS North East London ICB
- ☐ NHS North West London ICB
- ☐ NHS South East London ICB
- ☐ NHS South West London ICB
- ☐ NHS Bath and North East Somerset, Swindon and Wiltshire ICB
- ☐ NHS Bristol, North Somerset and South Gloucestershire ICB
- ☐ NHS Cornwall and the Isles of Scilly ICB
- ☐ NHS Devon ICB
- ☐ NHS Dorset ICB
- ☐ NHS Gloucestershire ICB
- ☐ NHS Somerset ICB
- ☐ NHS Bedfordshire, Luton and Milton Keynes ICB
- ☐ NHS Buckinghamshire, Oxfordshire and Berkshire West ICB
- ☐ NHS Frimley ICB
- ☐ NHS Hampshire and Isle of Wight ICB
- ☐ NHS Kent and Medway ICB
- ☐ NHS Surrey Heartlands ICB
- ☐ NHS Sussex ICB
- ☐ NHS Birmingham and Solihull ICB
- ☐ NHS Black Country ICB
- ☐ NHS Coventry and Warwickshire ICB
- ☐ NHS Herefordshire and Worcestershire ICB
- ☐ NHS Shropshire, Telford and Wrekin ICB
- ☐ NHS Staffordshire and Stoke-on-Trent ICB
- ☐ NHS Derby and Derbyshire ICB
- ☐ NHS Leicester, Leicestershire and Rutland ICB
- ☐ NHS Lincolnshire ICB
- ☐ NHS Northamptonshire ICB
- ☐ NHS Nottingham and Nottinghamshire ICB
- ☐ NHS Cambridgeshire and Peterborough ICB
- ☐ NHS Hertfordshire and West Essex ICB
- ☐ NHS Mid and South Essex ICB
- ☐ NHS Norfolk and Waveney ICB
- ☐ NHS Suffolk and North East Essex ICB
- ☐ NHS South Yorkshire ICB
- ☐ NHS Humber and North Yorkshire ICB
- ☐ NHS West Yorkshire ICB
- ☐ NHS North East and North Cumbria ICB
- ☐ NHS Cheshire and Merseyside ICB
- ☐ NHS Greater Manchester ICB
- ☐ NHS Lancashire and South Cumbria ICB
- ☐ NHS Ayrshire and Arran
- ☐ NHS Borders
- ☐ NHS Dumfries and Galloway
- ☐ NHS Fife
- ☐ NHS Forth Valley
- ☐ NHS Grampian
- ☐ NHS Greater Glasgow and Clyde
- ☐ NHS Highland
- ☐ NHS Lanarkshire
- ☐ NHS Lothian
- ☐ NHS Shetland
- ☐ NHS Tayside
- ☐ NHS Western Isles (Bòrd SSN nan Eilean Siar)
- ☐ Aneurin Bevan University Health Board
- ☐ Betsi Cadwaladr University Health Board
- ☐ Cardiff and Vale University Health Board
- ☐ Cwm Taf Morgannwg University Local Health Board
- ☐ Hywel Dda University Health Board
- ☐ Powys Teaching Health Board
- ☐ Swansea Bay University Local Health Board
- ☐ Belfast Health and Social Care Trust
- ☐ Northern Health and Social Care Trust
- ☐ South Eastern Health and Social Care Trust
- ☐ Southern Health and Social Care Trust
- ☐ Western Health and Social Care Trust
- ☐ Other

**D4 - How many patients do you have on your practice list?**

- ☐ Up to 5,000 patients
- ☐ 5,001-7,500 patients
- ☐ 7,501-10,000 patients
- ☐ 10,001-12,500 patients
- ☐ 12,501 patients or more

### **Ambient AI Scribes**

**Q1. Which ambient AI scribe(s) do you currently use?** (Select all that apply)

- ☐ Nuance Dragon Ambient eXperience (DAX)
- ☐ Lyrebird Health
- ☐ Heidi Health
- ☐ I-Scribe
- ☐ Tali.AI
- ☐ Suki AI
- ☐ DeepScribe
- ☐ Augnito Spectra
- ☐ Hepian Write
- ☐ HealthScribe (Amazon)
- ☐ Other (please specify): ____________

### **Errors and Error Rates**

**Q2a - Do you detect errors in the documentation generated by the ambient AI scribe(s)?**

- ☐ Never (No errors detected in any documentation)
- ☐ Rarely (Errors found in less than 10% of documentation)
- ☐ Sometimes (Errors found in 10-30% of documentation)
- ☐ Often (Errors found in 31-70% of documentation)
- ☐ Always (Errors found in nearly all documentation)

*If answered “rarely/sometimes/often/always” then answer:*

**Q2b - How would you rate the clinical significance of the majority of errors detected in relation to patient care?**

- ☐ Insignificant (No impact on patient care)
- ☐ Minor (Slight inaccuracies that don't affect patient care)
- ☐ Moderate (Errors requiring clarification but not affecting immediate patient care)
- ☐ Significant (Errors that could lead to inappropriate patient care if not caught)
- ☐ Critical (Errors that could result in serious harm to patient care if not detected)

*If answered 2b then answer;*

**Q2c - How frequently do you correct errors in AI-generated documentation?**

- ☐ Never
- ☐ Rarely (correct errors in less than 10% of documentation)
- ☐ Occasionally (correct errors in 10–30% of documentation)
- ☐ Often (correct errors in 31–70% of documentation)
- ☐ Almost always (correct errors in nearly all documentation)

*If answered 2b & c then answer;*

**Q2d - Are errors more common for specific patient groups?** (Check all that apply)

- ☐ Multiple patient’s/carers in the room
- ☐ Patients with complex medical histories
- ☐ Patients who speak a primary language other than English
- ☐ Patients with speech impairment
- ☐ Patients seen in noisy clinical environments
- ☐ Patients with cognitive impairment
- ☐ Older patients
- ☐ Other (please specify)
- ☐ No specific group

### **Litigation and Privacy Risks**

**Q3 - What is your level of concern regarding the medico-legal risks associated with using ambient AI scribes in medical documentation?**

- ☐ Not at all concerned
- ☐ Slightly concerned
- ☐ Moderately concerned
- ☐ Very concerned
- ☐ Extremely concerned

### **Perceptions of Burnout and Cognitive Burden**

Cognitive workload is the mental effort required to manage patient care, make decisions, and complete documentation. Ambient AI scribes aim to reduce this burden by automating note-taking.

**Q5 - How has using ambient AI scribes affected your cognitive workload during consultations?**

- ☐ Significantly decreased
- ☐ Somewhat decreased
- ☐ No change
- ☐ Somewhat increased
- ☐ Significantly increased

**Q6 - Overall, based on your definition of burnout, how would you rate your level of burnout?**

- ☐ I enjoy my work. I have no symptoms of burnout.
- ☐ Occasionally I am under stress, and I don’t always have as much energy as I once did, but I don’t feel burned out.
- ☐ I am definitely burning out and have one or more symptoms of burnout, such as physical and emotional exhaustion.
- ☐ The symptoms of burnout that I’m experiencing won’t go away. I think about frustration at work a lot.
- ☐ I feel completely burned out and often wonder if I can go on. I am at the point where I may need some changes or I may need to seek some help.

### **Time Spent on Documentation**

**Q7 - How has the use of ambient AI scribes affected the time you spend creating documentation?**

- ☐ Greatly decreased
- ☐ Slightly decreased
- ☐ No change
- ☐ Slightly increased
- ☐ Greatly increased

### **Perceptions of Documentation Quality**

**Q8 - In general, how do you perceive the quality of notes generated with ambient AI scribes?**

- ☐ Very poor (Significantly worse than standard medical documentation, requiring extensive revision)
- ☐ Poor (Below the standard of standard medical documentation, requiring moderate revision)
- ☐ Neutral (Comparable to standard medical documentation)
- ☐ Good (Better than standard medical documentation, requiring minimal revision)
- ☐ Very good (Consistently exceeds the quality of standard medical documentation, rarely needs revision)

### **Consent and Patient Interaction**

**Q9a - Do you routinely obtain patient consent before using an AI scribe during consultations?**

- ☐ Yes
- ☐ No

*If ‘Yes’ then asked*

**Q9b - What percentage of patients decline consent for the use of AI scribes?**

- ☐ 0%
- ☐ 1-10%
- ☐ 11-20%
- ☐ 21-30%
- ☐ 31% or more

### **Closing**

Thank you for completing the survey. Your feedback is invaluable in shaping the safe and effective use of AI tools in primary care documentation. If you have any additional comments, please include them below:

**Additional Comments:**

*Please add any comments about the topic or the survey (1–2 brief comments)*
