# Supplementary material for: Ambient AI in primary care: an exploratory mixed methods survey of UK general practitioners
Source: BMJ Health Care Inform. 2026 Jul 1;33(1):e101847. doi: 10.1136/bmjhci-2025-101847 (PMC13331185; doi:10.1136/bmjhci-2025-101847)
Supplement: online supplemental appendix 3 [file bmjhci-33-1-s003.docx]

**Appendix 3.** Additional analyses

**Table 1.** Comparison of proportion differences between GPs in the sample and those in the GMC Registry using chi-square goodness-of-fit test.

|  | **Sample**  (*n* = 1,003) | **GMC Registry**  (*n* = 68,692) | Analysis |
| --- | --- | --- | --- |
| **Gender** |  |  | χ^2^(1, 984) = 19.38, *p* < .001, Cramer’s V = .14 |
| Woman | 503 (51.1%) | 39,871 (58%) |  |
| Man | 481 (48.9%) | 28,821 (42%) |  |
| **Age** |  |  | N/A |
| 35 years or younger | 103 (10.3%) | 6,378 (9.3%) |  |
| 36 – 45 years | 349 (34.8%) | 25,731 (37.5%) |  |
| 46 – 55 years | 371 (37%) | 21,018 (30.6%) |  |
| 56 years or older | 180 (17.9%) | 15,565 (22.7%) |  |
| **Regional distribution** |  |  | χ^2^(10, 1829) = 11.98, *p* = .286, Cramer’s V = .03 |
| **England** | 826 (82.4%) | 57,362 (83.5%) |  |
| London | 116 (11.6%) | 10,190 (14.8%) |  |
| South West | 88 (8.8%) | 6,554 (9.5%) |  |
| South East | 131 (13.1%) | 9,087 (13.2%) |  |
| Midlands | 160 (15.9%) | 10,050 (14.6%) |  |
| East of England | 84 (8.4%) | 5,826 (8.5%) |  |
| North East & Yorkshire | 129 (12.9%) | 8,259 (12%) |  |
| North West | 118 (11.8%) | 7,396 (10.8%) |  |
| **Scotland** | 100 (10%) | 6,467 (9.4%) |  |
| **Wales** | 43 (4.3%) | 2,880 (4.2%) |  |
| **Northern Ireland** | 34 (3.4%) | 1,983 (2.9%) |  |

*Note*: Gender analysis excluded *‘Other’* (*n* = 1) and *‘Prefer not to answer’* (*n* = 18) responses, resulting in a sample of 984. Comparison of age was not carried out due to different age brackets in the survey and the GMC Registry (e.g., 35 – 44 years). Comparison of regional distribution was carried out with England regions, Scotland, Wales, and Northern Ireland as variable levels.

**Table 2.** Comparison of proportion differences between Ambient AI user and non-user GPs using two-proportion z-test.

|  | **Users**  (*n* = 141) | **Non-users**  (*n* = 862) | Difference | z-statistic | Unadjusted  *p* value | Bonferonni-adjusted  *p* value |
| --- | --- | --- | --- | --- | --- | --- |
| **Gender** |  |  |  |  |  |  |
| Woman | 45.4% | 51.9% | - 6.5% | - 1.22 | .223 | .446 |
| Man | 53.6% | 48.1% | + 5.5% | 1.22 | .223 | .446 |
| **Age** |  |  |  |  |  |  |
| 35 years or younger | 10.6% | 10.2% | + .4% | .16 | .876 | 1.000 |
| 36 – 45 years | 44% | 33.3% | + 10.7% | 2.47 | .014 | .054 |
| 46 – 55 years | 35.5% | 37.2% | - 1.8% | - .41 | .685 | 1.000 |
| 56 years or older | 9.9% | 19.3% | - 9.3% | - 2.68 | .008 | .03 * |
| **Role** |  |  |  |  |  |  |
| GP Partner or Principal | 53.9% | 40.6% | + 13.3% | 2.96 | .003 | .012 * |
| Salaried GPs | 36.9% | 38.5% | - 1.6% | - .37 | .711 | 1 |
| Locum GPs | 5 % | 17% | - 12% | - 3.67 | < .001 | = .001 ** |
| GP Registrar | 4.3% | 3.9% | + .3% | 0.18 | .861 | 1 |
| **GP practice size** |  |  |  |  |  |  |
| Up to 5,000 patients | 4.3% | 12.2% | - 7.9% | - 2.78 | .005 | .027 * |
| 5,001 – 7,500 patients | 13.4% | 15.2% | - 1.7% | - .53 | .595 | 1 |
| 7,501 – 10,000 patients | 19.9% | 20.3% | - .4% | - .12 | .903 | 1 |
| 10,001 – 12,500 patients | 17% | 17.2% | - .2% | - .04 | .966 | 1 |
| 12,501 patients or more | 45.4% | 35.1% | + 10.2% | 2.34 | .019 | .096 |

*Note*: Gender analysis excluded *‘Other’* (*n* = 1) and *‘Prefer not to answer’* (*n* = 18) responses, resulting in 138 users and 846 non-users.

**Table 3.** Percentage of patients who decline consent for the use of AI scribes.

|  | **Users**  (*n* = 141) |
| --- | --- |
| 0% | 52 (58.4%) |
| 1 — 10% | 27 (30.3%) |
| 11 — 20% | 3 (3.4%) |
| 21 — 30% | 1 (1.1%) |
| 31% or more | 6 (6.7%) |

*Note:* Scribes that were listed but received no votes: Augnito Spectra, Dragon Ambient eXperience (DAX), Heparin Write, HepianScribe, Lyrebird Health, Tali.AI.
